# Supplementary material for: Unveiling the Bioactive Potential of the Invasive Jellyfish Phyllorhiza punctata Through Integrative Transcriptomic and Proteomic Analyses
Source: Biomolecules. 2025 Aug 4;15(8):1121. doi: 10.3390/biom15081121 (PMC12383608; doi:10.3390/biom15081121)
Supplement: Supplementary file 1 [file biomolecules-15-01121-s001.zip › Suplementary Material/FileS3/PEPFOLD4/XP_065058281_4852/clusters.html]

 PEP-FOLD clusters


Download archive of all models  here   

|  |  |  |
| --- | --- | --- |
| # | file | sOPEP |
| # cluster 1 - Size: 2 |  |  |
|  | XP\_065058281\_4852-model1 | -6.79571 |
|  | XP\_065058281\_4852-model1.1 | -6.56498 |
| # cluster 2 - Size: 1 |  |  |
|  | XP\_065058281\_4852-model2 | -5.98715 |
| # cluster 3 - Size: 1 |  |  |
|  | XP\_065058281\_4852-model3 | -5.74741 |
| # cluster 4 - Size: 2 |  |  |
|  | XP\_065058281\_4852-model4 | -5.5165 |
|  | XP\_065058281\_4852-model4.1 | -5.35415 |
| # cluster 5 - Size: 1 |  |  |
|  | XP\_065058281\_4852-model5 | -5.49944 |
| # cluster 6 - Size: 1 |  |  |
|  | XP\_065058281\_4852-model6 | -5.49096 |
| # cluster 7 - Size: 1 |  |  |
|  | XP\_065058281\_4852-model7 | -5.38031 |
| # cluster 8 - Size: 4 |  |  |
|  | XP\_065058281\_4852-model8 | -5.24811 |
|  | XP\_065058281\_4852-model8.1 | -5.10527 |
|  | XP\_065058281\_4852-model8.2 | -5.0485 |
|  | XP\_065058281\_4852-model8.3 | -4.75885 |
| # cluster 9 - Size: 2 |  |  |
|  | XP\_065058281\_4852-model9 | -5.23863 |
|  | XP\_065058281\_4852-model9.1 | -4.99288 |
| # cluster 10 - Size: 5 |  |  |
|  | XP\_065058281\_4852-model10 | -5.19911 |
|  | XP\_065058281\_4852-model10.1 | -5.06363 |
|  | XP\_065058281\_4852-model10.2 | -5.0382 |
|  | XP\_065058281\_4852-model10.3 | -4.91815 |
|  | XP\_065058281\_4852-model10.4 | -4.83739 |
| # cluster 11 - Size: 6 |  |  |
|  | XP\_065058281\_4852-model11 | -5.15769 |
|  | XP\_065058281\_4852-model11.1 | -5.04312 |
|  | XP\_065058281\_4852-model11.2 | -4.96667 |
|  | XP\_065058281\_4852-model11.3 | -4.90919 |
|  | XP\_065058281\_4852-model11.4 | -4.86324 |
|  | XP\_065058281\_4852-model11.5 | -4.76293 |
| # cluster 12 - Size: 4 |  |  |
|  | XP\_065058281\_4852-model12 | -5.14293 |
|  | XP\_065058281\_4852-model12.1 | -4.86501 |
|  | XP\_065058281\_4852-model12.2 | -4.80755 |
|  | XP\_065058281\_4852-model12.3 | -4.79045 |
| # cluster 13 - Size: 1 |  |  |
|  | XP\_065058281\_4852-model13 | -5.12896 |
| # cluster 14 - Size: 2 |  |  |
|  | XP\_065058281\_4852-model14 | -5.11931 |
|  | XP\_065058281\_4852-model14.1 | -4.85058 |
| # cluster 15 - Size: 2 |  |  |
|  | XP\_065058281\_4852-model15 | -5.10607 |
|  | XP\_065058281\_4852-model15.1 | -4.97602 |
| # cluster 16 - Size: 6 |  |  |
|  | XP\_065058281\_4852-model16 | -5.10486 |
|  | XP\_065058281\_4852-model16.1 | -5.02192 |
|  | XP\_065058281\_4852-model16.2 | -5.01055 |
|  | XP\_065058281\_4852-model16.3 | -4.89846 |
|  | XP\_065058281\_4852-model16.4 | -4.8138 |
|  | XP\_065058281\_4852-model16.5 | -4.74238 |
| # cluster 17 - Size: 1 |  |  |
|  | XP\_065058281\_4852-model17 | -5.09626 |
| # cluster 18 - Size: 3 |  |  |
|  | XP\_065058281\_4852-model18 | -5.07707 |
|  | XP\_065058281\_4852-model18.1 | -4.87455 |
|  | XP\_065058281\_4852-model18.2 | -4.70176 |
| # cluster 19 - Size: 2 |  |  |
|  | XP\_065058281\_4852-model19 | -5.06545 |
|  | XP\_065058281\_4852-model19.1 | -5.03723 |
| # cluster 20 - Size: 2 |  |  |
|  | XP\_065058281\_4852-model20 | -5.06299 |
|  | XP\_065058281\_4852-model20.1 | -4.82632 |
| # cluster 21 - Size: 2 |  |  |
|  | XP\_065058281\_4852-model21 | -5.05419 |
|  | XP\_065058281\_4852-model21.1 | -4.87551 |
| # cluster 22 - Size: 2 |  |  |
|  | XP\_065058281\_4852-model22 | -5.04811 |
|  | XP\_065058281\_4852-model22.1 | -4.83928 |
| # cluster 23 - Size: 1 |  |  |
|  | XP\_065058281\_4852-model23 | -5.04347 |
| # cluster 24 - Size: 4 |  |  |
|  | XP\_065058281\_4852-model24 | -5.03037 |
|  | XP\_065058281\_4852-model24.1 | -5.02169 |
|  | XP\_065058281\_4852-model24.2 | -4.97127 |
|  | XP\_065058281\_4852-model24.3 | -4.7955 |
| # cluster 25 - Size: 5 |  |  |
|  | XP\_065058281\_4852-model25 | -5.02264 |
|  | XP\_065058281\_4852-model25.1 | -4.98845 |
|  | XP\_065058281\_4852-model25.2 | -4.97641 |
|  | XP\_065058281\_4852-model25.3 | -4.93275 |
|  | XP\_065058281\_4852-model25.4 | -4.89537 |
| # cluster 26 - Size: 3 |  |  |
|  | XP\_065058281\_4852-model26 | -5.01312 |
|  | XP\_065058281\_4852-model26.1 | -4.93552 |
|  | XP\_065058281\_4852-model26.2 | -4.90679 |
| # cluster 27 - Size: 1 |  |  |
|  | XP\_065058281\_4852-model27 | -4.99377 |
| # cluster 28 - Size: 3 |  |  |
|  | XP\_065058281\_4852-model28 | -4.97001 |
|  | XP\_065058281\_4852-model28.1 | -4.9511 |
|  | XP\_065058281\_4852-model28.2 | -4.91929 |
| # cluster 29 - Size: 3 |  |  |
|  | XP\_065058281\_4852-model29 | -4.94706 |
|  | XP\_065058281\_4852-model29.1 | -4.85541 |
|  | XP\_065058281\_4852-model29.2 | -4.7947 |
| # cluster 30 - Size: 3 |  |  |
|  | XP\_065058281\_4852-model30 | -4.94628 |
|  | XP\_065058281\_4852-model30.1 | -4.89579 |
|  | XP\_065058281\_4852-model30.2 | -4.84026 |
| # cluster 31 - Size: 2 |  |  |
|  | XP\_065058281\_4852-model31 | -4.93677 |
|  | XP\_065058281\_4852-model31.1 | -4.75612 |
| # cluster 32 - Size: 1 |  |  |
|  | XP\_065058281\_4852-model32 | -4.91904 |
| # cluster 33 - Size: 3 |  |  |
|  | XP\_065058281\_4852-model33 | -4.91597 |
|  | XP\_065058281\_4852-model33.1 | -4.8885 |
|  | XP\_065058281\_4852-model33.2 | -4.87952 |
| # cluster 34 - Size: 2 |  |  |
|  | XP\_065058281\_4852-model34 | -4.91134 |
|  | XP\_065058281\_4852-model34.1 | -4.73979 |
| # cluster 35 - Size: 1 |  |  |
|  | XP\_065058281\_4852-model35 | -4.90165 |
| # cluster 36 - Size: 1 |  |  |
|  | XP\_065058281\_4852-model36 | -4.89038 |
| # cluster 37 - Size: 1 |  |  |
|  | XP\_065058281\_4852-model37 | -4.87885 |
| # cluster 38 - Size: 4 |  |  |
|  | XP\_065058281\_4852-model38 | -4.86212 |
|  | XP\_065058281\_4852-model38.1 | -4.78454 |
|  | XP\_065058281\_4852-model38.2 | -4.72715 |
|  | XP\_065058281\_4852-model38.3 | -4.68887 |
| # cluster 39 - Size: 1 |  |  |
|  | XP\_065058281\_4852-model39 | -4.85856 |
| # cluster 40 - Size: 1 |  |  |
|  | XP\_065058281\_4852-model40 | -4.85586 |
| # cluster 41 - Size: 1 |  |  |
|  | XP\_065058281\_4852-model41 | -4.85245 |
| # cluster 42 - Size: 1 |  |  |
|  | XP\_065058281\_4852-model42 | -4.83225 |
| # cluster 43 - Size: 2 |  |  |
|  | XP\_065058281\_4852-model43 | -4.82358 |
|  | XP\_065058281\_4852-model43.1 | -4.69802 |
| # cluster 44 - Size: 1 |  |  |
|  | XP\_065058281\_4852-model44 | -4.80064 |
| # cluster 45 - Size: 1 |  |  |
|  | XP\_065058281\_4852-model45 | -4.78658 |
| # cluster 46 - Size: 1 |  |  |
|  | XP\_065058281\_4852-model46 | -4.61005 |
|  |  |  |
